# Broad Epigenetic Signature of Maternal Care in the Brain of Adult Rats

**DOI:** 10.1371/journal.pone.0014739

**Published:** 2011-02-28

**Authors:** Patrick O. McGowan, Matthew Suderman, Aya Sasaki, Tony C. T. Huang, Michael Hallett, Michael J. Meaney, Moshe Szyf

**Affiliations:** 1 Douglas Mental Health University Institute, Montreal, Quebec, Canada; 2 Sackler Program for Epigenetics and Developmental Psychobiology at McGill University, McGill University, Montreal, Quebec, Canada; 3 Centre for the Neurobiology of Stress, University of Toronto, Scarborough, Toronto, Ontario, Canada; 4 Department of Pharmacology and Therapeutics, McGill University, Montreal, Quebec, Canada; 5 McGill Centre for Bioinformatics, McGill University, Montreal, Quebec, Canada; 6 Singapore Institute for Clinical Sciences, Singapore, Republic of Singapore; 7 Experience-Based Brain and Biological Development Program of the Canadian Institute for Advanced Research, Toronto, Ontario, Canada; CNRS, France

## Abstract

**Background:**

Maternal care is associated with long-term effects on behavior and epigenetic programming of the *NR3C1* (*GLUCOCORTICOID RECEPTOR*) gene in the hippocampus of both rats and humans. In the rat, these effects are reversed by cross-fostering, demonstrating that they are defined by epigenetic rather than genetic processes. However, epigenetic changes at a single gene promoter are unlikely to account for the range of outcomes and the persistent change in expression of hundreds of additional genes in adult rats in response to differences in maternal care.

**Methodology/Principal Findings:**

We examine here using high-density oligonucleotide array the state of DNA methylation, histone acetylation and gene expression in a 7 million base pair region of chromosome 18 containing the *NR3C1* gene in the hippocampus of adult rats. Natural variations in maternal care are associated with coordinate epigenetic changes spanning over a hundred kilobase pairs. The adult offspring of high compared to low maternal care mothers show epigenetic changes in promoters, exons, and gene ends associated with higher transcriptional activity across many genes within the locus examined. Other genes in this region remain unchanged, indicating a clustered yet specific and patterned response. Interestingly, the chromosomal region containing the *protocadherin-α, -β,* and *-γ* (*Pcdh*) gene families implicated in synaptogenesis show the highest differential response to maternal care.

**Conclusions/Significance:**

The results suggest for the first time that the epigenetic response to maternal care is coordinated in clusters across broad genomic areas. The data indicate that the epigenetic response to maternal care involves not only single candidate gene promoters but includes transcriptional and intragenic sequences, as well as those residing distantly from transcription start sites. These epigenetic and transcriptional profiles constitute the first tiling microarray data set exploring the relationship between epigenetic modifications and RNA expression in both protein coding and non-coding regions across a chromosomal locus in the mammalian brain.

## Introduction

The quality of parental care has a broad impact on mental health, including the risk for psychopathology [1], [2], [3], [4], [5]. Studies in the rat directly link the maternal care environment to long-term effects on neural systems that regulate stress [6], [7] emotional function[8], [9], learning and memory [10], [11], [12] and neuroplasticity [10], [13], [14], [15]. Naturally occurring variations in maternal care in the first week of life in rats are associated with changes in brain and behavior that persist until adulthood [16]. These effects are reversed by cross-fostering, [7], [9] demonstrating a causal link between maternal care and gene expression programming.

In rats and humans, there is evidence that changes in gene expression as a function of early care are at least partly regulated by epigenetic mechanisms [6], [17], [18]. In rats, variations in maternal care in the first week of life are associated with alterations in DNA methylation and H3K9 acetylation of the *NR3C1* promoter region, and gene expression of the GR1_7_ splice variant of the *NR3C1* gene in the hippocampus of adult offspring [6]. There is evidence that the expression of hundreds of additional genes in adult rats changes in response to differences in maternal care [19]. Some of these changes in gene expression can be reversed by pharmacological alterations of chromatin structure by the histone deacetylase inhibitor Trichostatin A (TSA) and the methyl donor L-methionine [19], [20]. The fact that the methyl donor L-methionine inhibits some of the genes influenced by maternal behavior supports the involvement of either DNA or histone methylation. The fact that a large number of genes are responsive to the effects of TSA and L-methionine implies that the epigenetic regulation of gene expression as a function of maternal care may be extensive. In the present study, we test this hypothesis by examining epigenetic and transcriptional changes associated with naturally occurring differences in maternal care.

We obtained hippocampal samples from the adult offspring of rat mothers that differed in the frequency of pup licking/grooming in the first week of life (i.e. High vs Low LG adult offspring) and performed an analysis of DNA methylation, H3K9 acetylation and gene expression of a contiguous 7 million base pair region of rat chromosome 18 containing the *NR3C1* gene at 100 bp spacing. To our knowledge, these epigenetic and transcriptional profiles constitute the first tiling microarray data set exploring the relationship between epigenetic modifications and RNA expression in both protein coding and non-coding regions across a chromosomal locus in the mammalian brain.

## Results

### Validation of microarray results

**Figure 1:**
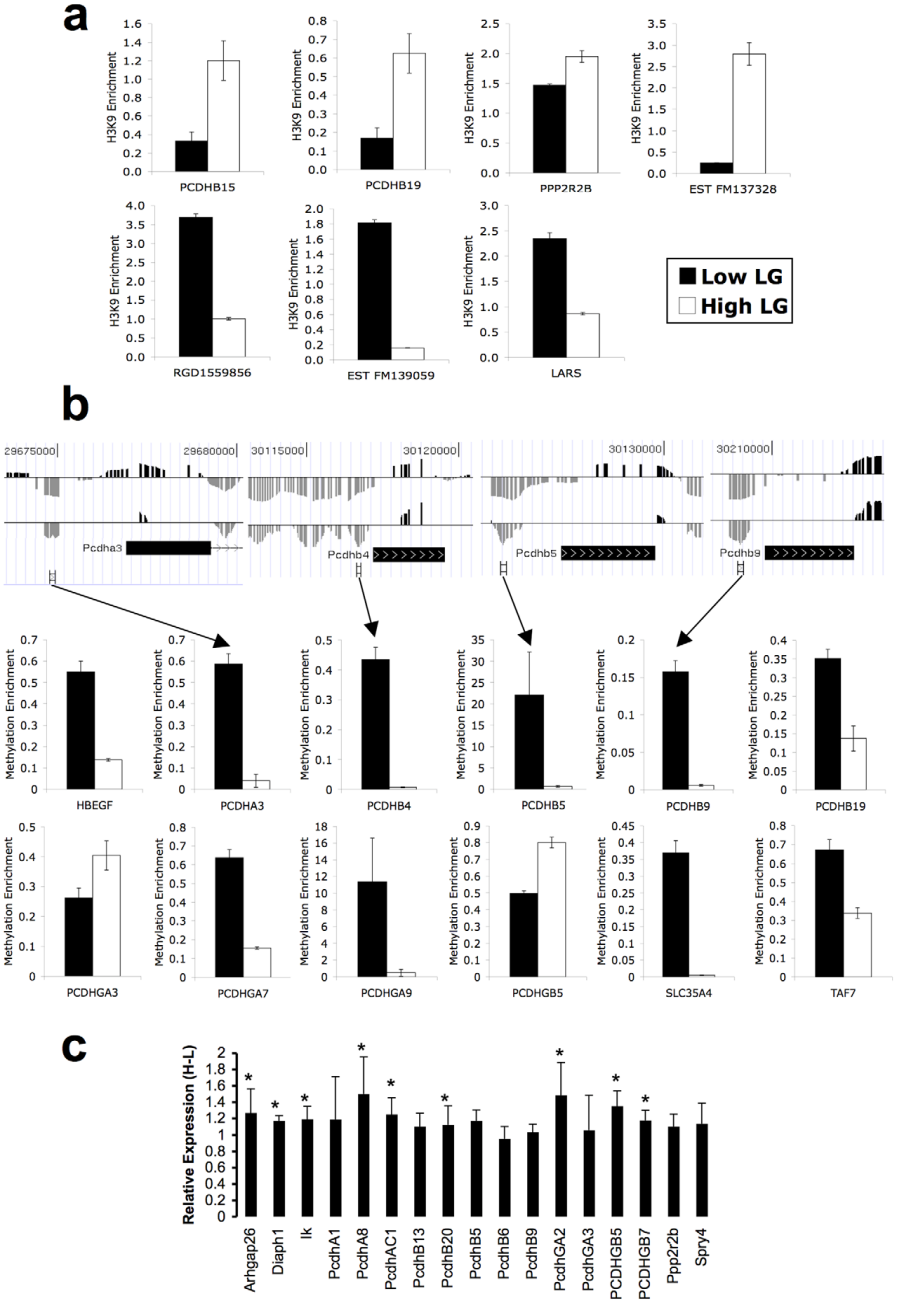
Microarray validation. (**a**) H3K9 acetylation differences between High (white bars) and Low LG (black bars) adult offspring validated by qCHIP (see **Methods**). (**b**) (upper) DNA methylation differences between High and Low LG adult offspring detected by microarray analysis (H–L), showing gene location, and region analyzed. (lower) DNA methylation differences validated in the same manner as for H3K9 acetylation. (**c**) Gene expression differences between High LG and Low LG adult offspring (* = *P*<0.05). All real-time PCR reactions were performed in triplicate and results are displayed as mean +/− SEM.

To validate signals observed on our microarray and differences between High and Low LG offspring, we quantified changes in H3K9 acetylation, DNA methylation, and transcription. H3K9 acetylation differences in 7 regions (**Fig. 1a**) and DNA methylation differences in 12 regions (**Fig. 1b**) were validated by quantitative PCR (qChIP – see **Methods** for details; [21]). Levels of DNA methylation validated by qChIP correlated significantly with levels of enrichment detected by microarray (*R* = 0.38, *P* = 0.0029 by Pearson's correlation; **Fig. S1**). DNA methylation differences were further confirmed for four genes by sequencing sodium bisulfite converted DNA (**Fig. S2**). False positives due to DNA polymorphism rather than differential methylation were ruled out for 12 regions (those validated by qChIP above) via DNA sequencing (data not shown). Of nine genes showing significant differences in gene expression between High and Low LG offspring, all were significantly more expressed among High LG offspring (**Fig. 1c**).

As a further method of validating our microarrays, we compared our average observed levels of transcriptional and epigenetic signals to previously described signals within specific gene elements across the entire locus profiled. To do so, we examined the absolute levels of transcription, histone acetylation and DNA methylation for all subjects combined (**Fig. S3**), and compared them to previously published relationships between levels of gene expression, DNA methylation, and histone acetylation across 5′ regulatory regions, exons, and introns. First, previous studies have indicated that much of the genome is actively transcribed [22] but that levels of transcription are generally higher within annotated exons relative to other regions. As expected, inside exons we observed significantly higher transcription than the overall levels of transcription throughout all regions in the locus (*P* = 1.47×10^−155^ by Student's T-test, *P* = 0 by Wilcoxon Rank Sum test). In contrast, we observed levels of transcription just upstream of genes (−1800 bp to transcription start site) and in intronic regions that were indistinguishable from the baseline. These data indicate that the signals observed by our microarray accurately detect known transcribed regions. Second, many previous studies in a variety of cell types have shown that active transcription is associated with low levels of DNA methylation in the 5′ ends of genes [23]. CpG islands also show lower than average levels of DNA methylation compared to other genomic regions [24]. As expected, we observed lower DNA methylation levels in 5′ gene ends (*P* = 1.34×10^−78^ by Wilcoxon Rank Sum test) and within CpG islands (*P* = 7.15×10^−200^ by Wilcoxon Rank Sum test) than the overall levels of methylation across the locus (**Fig. S3b–c**). Third, actively transcribed genes have been associated with reduced nucleosome occupancy near transcription start sites [25], [26], [27]. We similarly found lower H3K9 acetylation levels in 5′ gene ends (*P* = 9.22×10^−47^ by Wilcoxon Rank Sum test; **Fig. S3b**). Computational prediction of nucleosome density from DNA sequence [28] showed a significant correlation between nucleosome position and H3K9 acetylation levels observed by microarray (*R* = 0.2, *P* = 2.2×10^−16^ by Pearson's correlation; **Fig. S4**). These observations of lower absolute levels of DNA methylation with CpG islands and higher levels of transcription within exons associated with lower DNA methylation and H3K9 acetylation levels in 5′ gene ends indicate that our epigenetic and transcription microarray results conform to previously published data in other genomic loci.

### The pattern of the epigenetic and transcriptional response to maternal care across the *NR3C1* gene locus

**Figure 2:**
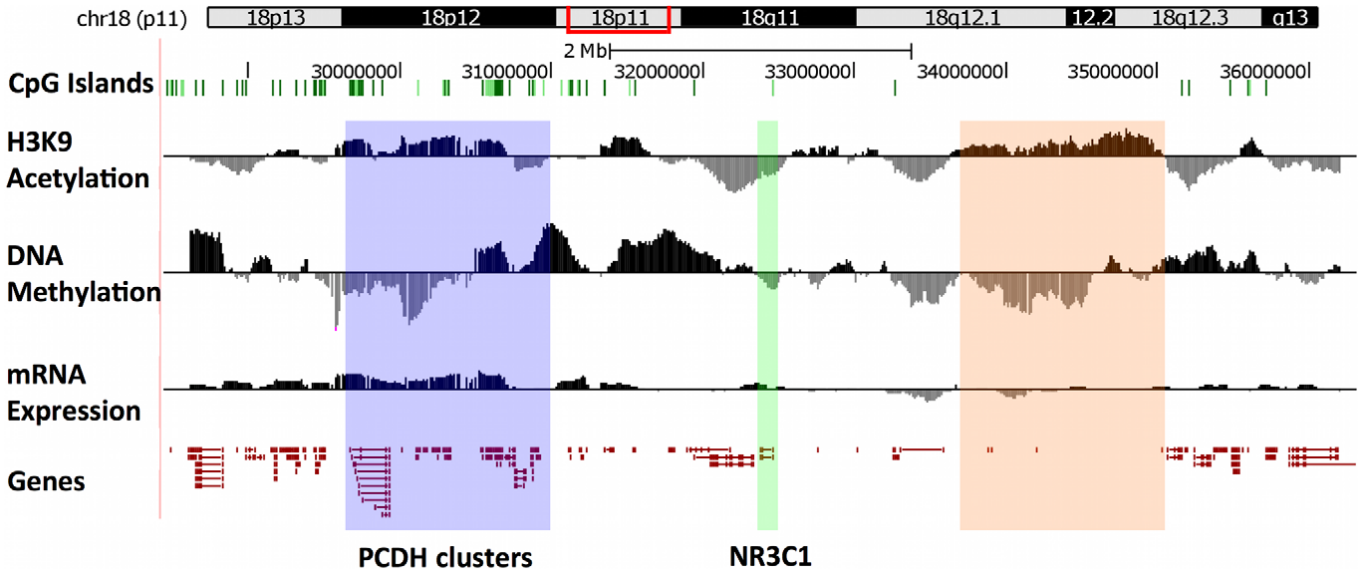
The pattern of H3K9 acetylation, DNA methylation, and gene expression among High and Low LG adult offspring across ∼7MB of chromosome 18. Tracks show CpG Islands, differences in H3K9 acetylation, DNA methylation and gene expression between High (black) and Low LG (grey) adult offspring (H–L) and the locations of known genes (red) across the chromosomal locus (see **Methods S1**). Highlighted regions show the location of the *NR3C1* gene (green), Protocadherin gene clusters (blue) and a large mainly intergenic region (orange).

A “large-scale” view of the entire locus as a whole revealed a widespread but patterned response to maternal care among High and Low LG adult offspring (High – Low; **Fig. 2**). We observed peaks and valleys of H3K9 acetylation and DNA methylation levels throughout a number of regions, suggesting a widespread epigenomic response to variations in maternal care. The response to maternal care is not evenly distributed, with many sequences showing little or no response and clustered regions showing enhanced responses. In total, we found significant differential DNA methylation in 1413 probes and significant differences in H3K9 acetylation in 713 probes out of 44000 probes covering the region. Variations in epigenetic signaling across the locus appear within annotated genic regions (e.g., **Fig. 2** – see blue highlight), and also in regions where no gene is annotated (e.g., **Fig. 2** – see orange highlight). Transcriptional differences are similarly widespread (**Fig. 2** – see expression track). These results suggest that some but not all regions are associated with changes in epigenetic signaling associated with differences in maternal care, with broad epigenetic changes apparent within both genic and inter-genic areas.

### Localization of broad epigenetic changes to gene regulatory and transcriptional elements

**Figure 3:**
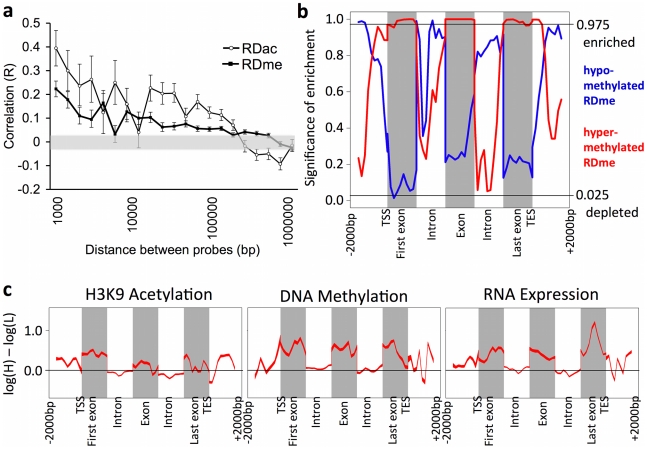
Regional variations in differences in histone acetylation, DNA methyation and gene expression between High and Low LG adult offspring. (**a**) The Pearson correlation of DNA methylation and H3K9 acetylation differences between the High and Low LG adult offspring for pairs of probes located at varying distances from each other. Error bars show 95% confidence intervals for the correlation values. Grey highlight shows the 95% confidence interval for correlations obtained from randomly selected probe pairs. (**b**) Enrichment of RDme (Regional Differences in DNA methylation) between High and Low LG adult offspring across all genes from the 5′ region to the 3′ region. Enrichment is quantified as increased frequency of RDme in a given gene region (number of RDme/bp). Significance is the quantile of this enrichment with respect to the distribution of randomly positioned RDme. A quantile above 0.975 indicates significant enrichment, and a quantile below 0.025 indicates significant depletion at the *P* = 0.05 level. Quantiles of hyperacetylated RDac/hypermethylated RDme in High compared to Low LG offspring (red) and quantiles of hypoacetylated RDac/hypomethylated RDme (blue) are shown. (**c**) Mean differences across all probes in DNA methylation, H3K9 acetylation and RNA expression levels between High LG and Low LG adult offspring are shown across all genes from the 5′ region to the 3′ region, with significant differences indicated by non-zero values. Line thickness denotes SEM.

To index broad epigenetic changes observed across the locus, we defined a Regional Difference in DNA methylation and a Regional Difference H3K9 acetylation (RDme and RDac, respectively) as a statistically significant difference between High LG and Low LG offspring of at least 1000 bp containing at least one statistically significant probe per 1000 bp (see **Methods S1** for details). Across the entire locus, we identified 723 RDme of which 373 are significantly hypermethylated and 350 are hypomethylated in High relative to Low LG offspring. We similarly identified 471 RDac of which 204 are hyperacetylated and 267 are hypoacetylated. We found that these broad epigenetic differences associated with maternal care are significantly co-localized within the locus, and were positively correlated at distances over 100 Kb (**Fig. 3a**). The data suggest that clustering of differentially methylated and acetylated regions is not exclusive to pathological responses under extreme selection as is the case in cancer but includes epigenetic responses to natural variations in maternal care, and may be characteristic of naturally occurring epigenetic responses.

We next examined the localization of broad differences in H3K9 acetylation, DNA methylation (i.e., RDac and RDme) and transcription with respect to the physical anatomy of genes within the locus. Gene regulatory elements, including transcription start sites, 5′ and 3′ gene ends, and CpG islands are typical regions of interest in studies of gene regulation by epigenetic mechanisms. We found no evidence of a relationship between CpG density and the presence of RDme (*P* = 0.53 by Wilcoxon rank sum test), indicating there is no difference between CpG islands and other regions with respect to the presence of RDme. RDme/ac overlapped the transcription start sites of some but not all genes, indicating specificity in epigenetic signaling within the locus. Seventy-seven transcription start sites in 69 genes contain RDme while 127 transcript start sites in 94 genes do not contain RDme. Similarly, 37 transcription start sites in 32 genes contain RDac while 167 transcription start sites in 131 genes do not contain RDac. There was a significant enrichment of hyperacetylated RDac (regions in which the high maternal care group has higher acetylation levels) inside exons, particularly the first and last exons (*P* = 0.0014 and *P* = 0.0088, respectively; permutation test), and a significant depletion of hypoacetylated RDac inside the first and last exons (*P* = 0.0002 and *P* = 0.19, respectively; permutation test). RDac are relatively depleted in the 5′ and 3′ ends of genes (*P* = 0.02 by permutation test), likely reflecting the aforementioned depletion of nucleosomes at these sites in actively transcribed genes. In contrast, RDme co-localize in regulatory elements, particularly in the 5′ and 3′ ends of genes (*P* = 0.0032 by permutation test). Hypermethylated RDme (regions that are more methylated in the high maternal group than in the low maternal care group) are significantly enriched inside both the first and last exons of genes (*P* = 0.0008 and *P* = 0.004, respectively; permutation test) whereas hypomethylated RDme are significantly depleted inside the first exon (*P* = 0.0022; permutation test; **Fig. 3b** – red for hypermethylated RDme and blue for hypomethylated RDme). In addition, we observed an enrichment of hypomethylated RDme upstream of the TSS (*P* = 0.02; **Fig. 3b** – blue line). These data showing an enrichment of hyperacetylated RDac and hypermethylated RDme within exons and an enrichment of hypomethylated RDme in regulatory elements are consistent with previous data in cancer cells showing high exonic H3K9 acetylation [25], [26], [27] and DNA methylation [29] and low promoter DNA methylation associated with actively transcribed genes.

Next, we performed an analysis of probe-level changes in epigenetic and transcriptional signaling as an alternative method to compare to previous studies in cancer. We compared probe-level differences in H3K9 acetylation, DNA methylation, and RNA transcription to (1) identify whether our data show a similar correspondence between higher levels of transcription observed in the High LG offspring and epigenetic changes we expect based on studies in cancer cells and (2) examine whether the observed patterns at the level of individual probes are indicative of our analyses of RDme and RDac. **Figure** **3c** shows differences in H3K9 acetylation, DNA methylation, and RNA expression, with non-zero values indicating significant differences between High and Low LG offspring and line thickness denoting the standard error of the mean. In agreement with previous studies in cancer and the analyses of RDac/me above, H3K9 acetylation levels are significantly higher inside exons of the High LG offspring compared to the Low LG offspring, particularly the first and last exons (**Fig. 3c** – left panel). DNA methylation differences between the groups are, on average, significantly higher within exons among High LG offspring compared to Low LG offspring (**Fig. 3c** – middle panel). Expression differences inside exons indicate that High LG offspring show, on average, significantly higher RNA expression within annotated genes among High LG offspring compared to Low LG offspring (**Fig. 3c** – right panel). These data confirm previously published observations in cancer cells showing an association of actively transcribed genes with hyperacetylation and high methylation within exons [25], [26], [27], [29]. Taken together, these analyses within the regulatory and transcriptional elements of the genes in the locus are consistent with an observed significantly higher overall transcriptional activity among High LG adult offspring.

### NR3C1 gene and identification of novel candidate genes regulated by maternal care

**Figure 4:**
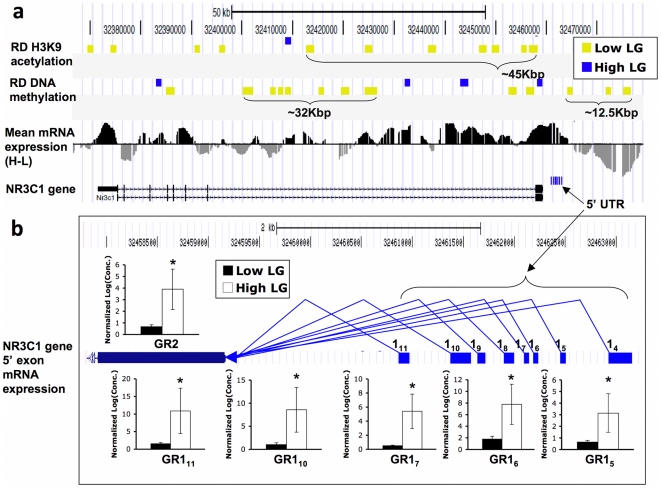
Epigenomic neighborhood of the glucocorticoid receptor gene. (**a**) Track of the glucocorticoid receptor gene, *NR3C1*, showing examples of large RDme/ac throughout the 5′ end and intron of the gene. Individual tracks, from the top to the bottom, show locations of hyperacetylated (blue) and hypoacetylated (yellow) RDac in High relative to Low LG adult offspring, RDme displayed in the same manner, mean gene expression, and the location of the *NR3C1* gene. (**b**) Schematic representation splice variant assembly of 5′ untranslated elements as well as the first coding exon (GR2) of the *NR3C1* gene, along with gene expression differences between High and Low LG adult offspring (* = *P*<0.05). Each real-time PCR reaction was performed in triplicate and results are displayed as mean +/− SEM.

We previously reported that NR3C1 gene expression and H3K9 acetylation were increased and DNA methylation was decreased in the promoter of the exon 1_7_ splice variant among High LG offspring compared to Low LG offspring [6], [7]. Using our comprehensive coverage of the entire NR3C1 locus we were able to identify additional novel regions of differential transcription, DNA methylation and histone acetylation in response to maternal care. We observed a number of RDme and RDac co-localized within intronic regions and upstream of the promoter region within the *NR3C1* gene (**Fig. 4a**). The *NR3C1* gene is known to contain at least 11 untranslated 5′ exon 1 splice variants that encode a common protein via a splice acceptor site on the exon 2. In this way, tissue-specific expression of NR3C1 is regulated by alternative splicing [30]. Our gene expression data agrees with previous studies showing that the expression of the GR exon 1_7_ splice variant as well as that of exon 2 is increased in High LG offspring ([6], [7]; **Fig. 4b**). Furthermore, we also detected increased transcription among High LG offspring in each of the exon 1 splice variants known to be expressed in the hippocampus: GR1_5_, GR1_6_, GR1_7_, GR1_10_, and GR1_11_ ([30]; **Fig. 4b**). These results suggest that broad epigenetic differences within the NR3C1 gene as well as the coordinated expression pattern of NR3C1 splice variants may be involved in the response to maternal care.

In addition to the *NR3C1* gene, a number of other genes show a significant number of RDme and are induced in response to differences in maternal care. We observed a broad genomic region that shows a cluster-wide response in DNA methylation and expression and exhibits the highest number of RDme relative to other regions in the locus: the -α, -β, and -γ protocadherin (Pcdh) gene clusters (82 of 696 RDme; *P* = 0.006, permutation test). Among Low LG offspring, we observed a significant enrichment for hypermethylated RDme across the entire Pcdh gene cluster (45 of a total of 350 RDme hypermethylated in Low LG offspring were found within the Pcdh gene clusters; *P* = 0.01, permutation test).

**Figure 5:**
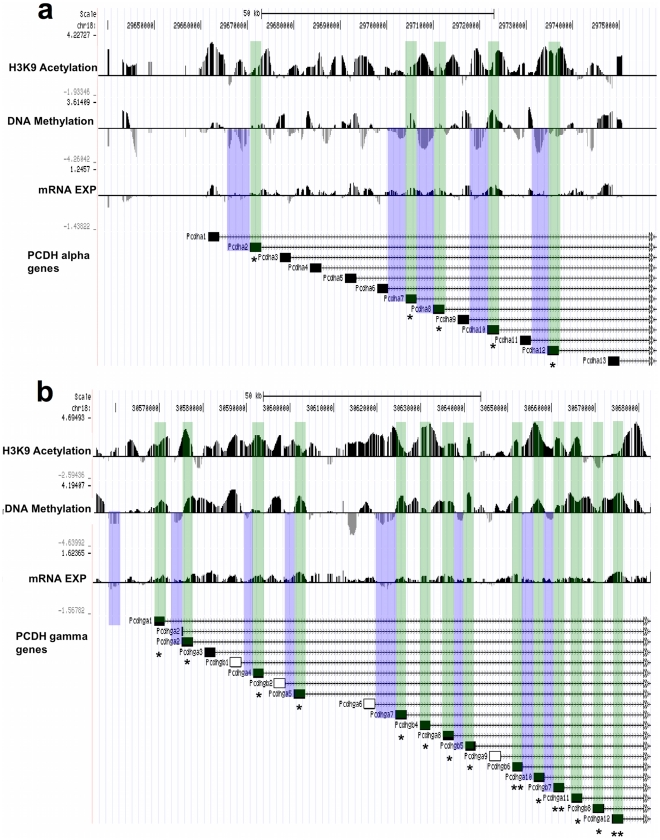
Epigenomic neighborhoods of the first exons in protocadherin gene clusters. Genes with hypomethylated 5′ gene ends (blue), hypermethylated and hyperacetylated exons (green) and significantly greater gene expression among High compared to Low LG adult offspring (H–L) are shown for (**a**) protocadherin-α, (**b**) protocadherin-γ gene clusters. Gene expression differences of genes surveyed by quantitative RT-PCR (filled boxes; ** = *P*<0.01, * = *P*<0.05) are shown relative to the location of other nearby Pcdh genes within each cluster (unfilled boxes).

Pcdh genes are predominantly expressed in neurons at synaptic junctions, and the assembly of these cell surface proteins is regulated by differential promoter activation and alternative pre-mRNA splicing [31]. Although the mechanisms underlying differential promoter activation are not well understood, promoter DNA methylation and histone acetylation play a role in Pcdh gene silencing [32], [33]. Consistent with this hypothesis, Pcdh gene expression induced in response to High LG maternal care is accompanied by higher in exonic H3K9 acetylation and DNA methylation (*P*<1×10^−300^ for both by Wilcoxon rank sum test) and lower proximal promoter DNA methylation in a majority (17 of 23, or 74%) of Pcdh genes showing a significant increase in expression among High LG compared to Low LG offspring (**Fig. 5**). High LG offspring show a significant increase in transcription in 20 Pcdh of 33 genes profiled within the Pcdh gene clusters (**Table S1**). Taken together, these results showing a transcriptional and epigenetic response to maternal care across the Pcdh gene family suggest that the epigenomic response to maternal care may act coordinately on a family of genes localized in the same broad genomic region.

## Discussion

The quality of maternal care in rodents has a widespread impact on phenotype that persists into adulthood, providing a model to study epigenetic mechanisms mediating the impact of the early life social environment on health later in life [6]. In this study, we asked whether our traditional approach examining the regulatory elements of candidate genes reflects the totality of the epigenetic response to naturally occurring environmental stimuli. By extending our analysis beyond the predicted boundaries of the *NR3C1* gene using high-density coverage of megabases of sequence, we investigated whether changes are limited to a small number of candidate genes, whether the changes are limited to 5′ regulatory regions and whether they are exclusive to regions encoding mRNAs.

We found non-random patterns of epigenetic and transcriptional alterations in a number of genes in association with differences in maternal care (**Fig. 2**). The specificity of this pattern is further underscored by the fact that both increased and decreased peaks of acetylation and DNA methylation are observed throughout the region. However, the response is gene-specific, as not all genes appear to respond to differences in maternal care (**Table S1**). Differences in broad epigenetic marks co-cluster over large distances (**Fig. 3a**), supporting previous work in cancer cells and suggesting the possibility of widespread epigenetic effects on multiple genes in the same genomic regions in response to maternal care. Analysis of our data with respect to protein-coding genes reveals expected relationships between epigenetic marks and gene expression levels. Increased transcription is associated with decreased 5′ DNA methylation and increased exonic H3K9 acetylation and DNA methylation (**Fig. 3b–c**). Previous studies have examined the relationships between differences in DNA methylation and histone acetylation and gene expression in on/off states of gene expression activity, as seen in cancer and cellular differentiation paradigms[23], [25], [26], [27], [29]. Our data suggest that the *modulation* of gene expression in response to environmental stimuli follows the same rules.

Our approach using high-density tiling microarrays also provided us with a “macroscopic” perspective of the epigenetic and transcriptional responses to maternal care. By zooming out of the specific suspected regions, we discovered differentially-methylated and acetylated regions that span large domains of sequence in the vicinity of the *NR3C1* gene (**Fig. 4a**). Among adult offspring of animals that had received relatively low levels of maternal care, we identified several hypermethylated RDme and hyperacetylated RDac upstream of the 5′ NR3C1 exon variants as well as in intronic regions, where transcription was also detected. These results suggest the possible involvement of non-coding RNAs and alternative splice variants in response to maternal care. Future studies are required to determine whether these broad regions that are differentially methylated in response to maternal care regulate NR3C1 expression.

Although when examined individually, different regions exhibit highly specific responses (**Fig. 4b**; **Fig. 5a–b**), large-scale patterns emerge when we use a “macro” view of the entire chromosomal region (**Fig. 2**). Both increased and decreased peaks of acetylation and DNA methylation are observed throughout the region. In addition, of the 29 transcripts showing statistically significant differences in transcription, all are significantly more expressed among High LG adult offspring (**Table S1**). These data indicate specificity in transcriptional changes at the single gene level as well as an overall common response at a “large-scale” level consisting of many neighboring genes. The fact that the observed response is a result of a naturally-occurring variation in maternal care rather than average “static” levels of histone acetylation and DNA methylation points to the possibility that a long-range coordinated regulation of genome function may play a role in the long-term programming of the genome.

One possible role for clustering of epigenetic responses across wide areas is the coordinate regulation of a large group of functionally related genes. We discovered that the expression of a large cluster of the Pcdh genes is coordinately regulated with respect to maternal care. Remarkably, the increase in gene expression in the High maternal care group spans genes within each of the Pcdh gene clusters (**Fig. 1c**; **Fig. 5a–b**). This family of genes correspondingly contains a significant overrepresentation of differentially methylated regions. It is interesting to speculate that the Pcdh gene family may have evolved though gene duplication as a class of functionally-related genes under coordinate epigenetic regulation. Indeed, coordinated silencing of the Pcdh family of genes was seen in cancer [34]. However, in cancer, processes related to cell-selection might be involved in a progressive spreading of DNA methylation [35], [36], [37].

We recognize that we do not yet know whether differences in Pcdh gene expression play a role in the effects of maternal care on brain function in offspring. Future studies are needed to examine the consequences of the epigenetic regulation of Pcdh gene expression for the regulation of Pcdh protein and downstream functional effects. Pcdh genes are preferentially expressed in neurons, including the hippocampus, and regulate synaptic development and function [38]. Pcdh-α gene expression during rodent neural development is highest in early postnatal life (until PD21), when it is involved in specifying the innervation of serotonergic neurons in the hippocampus [39]. Studies indicate enduring influences of differences in maternal care on hippocampal neuroplasticity, including effects on LTP [40], [41] and synaptic morphology [42], [43]. Indeed, a rich literature suggests widespread effects of the prenatal and postnatal environment on the developing brain (for reviews see [44], [45]). For example, whereas other maternal factors such as maternal stress during pregnancy induce long-term influences on behavior, including hippocampally-mediated fear conditioning and spatial learning, adoption studies show that postnatal maternal care can reverse these effects [11], [13], [46]. Both hippocampal synaptic density and LTP as well as contextual fear conditioning and spatial learning vary as a function of maternal care in the rat [10], [15]. It is interesting to speculate that differences in Pcdh gene regulation may be functionally relevant for hippocampal development.

The mechanisms responsible for this coordinated epigenomic response and its maintenance into adulthood are unknown. We observed a broad epigenomic response associated with an extensive difference in gene expression. These broad epigenomic and transcriptome changes occurred not in response to disease (e.g. cancer) [34] or artificial interventions (e.g., gene knock-out or exposure to toxins), but in the context of a natural variation in maternal behavior. Although the changes are broad, not all genes are affected. The specificity of the response and its pattern are consistent with the hypothesis that the epigenetic response is indeed a biological signal. Our data suggest that epigenetic variations in the context of early life environment variations and perhaps other environmental influences involve coordinate changes in gene-networks rather than dramatic changes in a single or few genes. Our data also suggest that this response may involve more than protein coding mRNAs. Our traditional approaches to examine relationships between epigenetic regulation, gene function and phenotype were developed to examine changes within genetic elements defined apriori (promoters, exons, 3′ gene ends) in single or few candidate genes. If, in addition, the response to an environmental stimulus such as maternal care involves more widespread or coordinated changes across multiple genomic regions, new experimental approaches are needed to examine the contribution of these changes to the ultimate phenotype. Our data suggest multiple levels of variations in DNA methylation and H3K9 acetylation, from site-specific gene-specific responses as previously reported [6], [17] to the regional responses shown in this study. Although future experiments are needed to address the relative role of “micro” and “macro” epigenetic responses, these data suggest that the broad epigenetic regulation of gene expression may form part of a coordinated response to early maternal care.

## Materials and Methods

### Ethics Statement

All procedures were performed according to guidelines developed by the Canadian Council on Animal Care and the protocol was approved by the McGill University Animal Care Committee, permit number 3284.

### Subjects and tissue preparation

Three to 4 animals per group were used in all microarray and quantitative immunoprecipitation experiments. An additional cohort of 8 animals per group was used for gene expression analysis. A maximum of 2 animals from any one litter were used, to control for possible effects attributable to variation between litters rather than variation as a function of High and Low LG. The animals were Long-Evans hooded rats born in our colony originally obtained from Charles River Canada (St. Constant, Québec). Maternal behavior was scored by using a version of the procedure described elsewhere[16]. Hippocampal tissue was dissected from 90-day-old (adult) male High and Low LG offspring and stored at −80°C. Genomic DNA extraction (DNeasy, Qiagen) and quantification (Nanodrop ND-1000 spectrophotometer, Thermo Scientific) as well as RNA extraction (RNeasy plus, Qiagen) and quality assessment (Bioanalyzer 2100, Agilent) were performed according to the manufacturer's protocol (see **Methods S1** for details).

### Chromatin/DNA immunoprecipitation and microarray hybridization

The procedure for methylated DNA immunoprecipitation was adapted from previously published work,[47], [48], [49] and H3K9 acetylation ChIP assays[50] were performed using the ChIP assay kit protocol (06-599, Upstate Biotechnology), as previously described[6]. The amplification (Whole Genome Amplification kit, Sigma) and labeling reaction (CGH labeling kit, Invitrogen), and all the steps of hybridization, washing and scanning were performed following the Agilent protocol for chip-on-chip analysis (see **Methods S1** for details). Three animals per group were used in the immunoprecipitation microarray experiments, and microarrays were hybridized in triplicate for each sample.

### cDNA microarray hybridization

RNA spike-in controls (Agilent) were added to RNA prior to generating cDNA. The cDNA was amplified and labeled with Cy3 or Cy5 (GE Healthcare) according to manufacturer's instructions (Fairplay III, Agilent; See **Methods S1** for details). Four animals per group were used for the gene expression microarrays, and a dye-swap experiment was performed for each subject in duplicate.

### Microarray design and analysis

Custom 44 K tiling arrays were designed using eArray (Agilent technologies). Probes of approximately 55 bp were selected to tile all unique regions within approximately 3.5 MB upstream and downstream of the *NR3C1* gene described in Ensembl (version 44) at 100 bp-spacing. Probe intensities were extracted from hybridization images using Agilent's Feature Extraction 9.5.3 Image Analysis Software and analyzed using the R software environment for statistical computing[51]. Log-ratios of the bound (Cy5) and input (Cy3) microarray channel intensities were computed for each microarray. Each microarray was normalized using quantile-normalization[52] assuming an identical overall distribution of measurement across all samples. Gene expression levels were estimated as the mean probe values across exons. DNA methylation and H3K9 acetylation levels at genomic locations were estimated using a Bayesian convolution algorithm to incorporate probe values from nearby probes[53]. Gene expression differences associated with maternal care were obtained using RMA[54] applied to sample probe values inside exons. RDme/ac were identified by computing a modified t-statistic for each probe and then significant levels of agreement across 1000 bp regions. Enrichment of RDac and RDme was determined by comparing base-pair overlap of these regions with overlap of randomly selected RDac/RDme (see **Methods S1** for details). All microarray data are MIAME compliant and the raw data have been deposited in Gene Expression Omnibus (GEO) at NCBI (www.ncbi.nlm.nih.gov/geo/), accession number pending.

### Quantitative real-time PCR of immunoprecipitated samples (qCHIP)

Gene-specific real-time PCR validation of microarray was performed in an identical manner for H3K9 acetylation and DNA methylation enrichment[21] for the same subjects used for microarray experiments (n = 3/group; see **Methods S1** for details). Relative enrichment of triplicate reactions were determined as a ratio of the crossing point threshold (Ct) of the amplified immunoprecipitated fraction (with either anti-histone H3K9 acetylation or anti 5-meC antibody) over the Ct of the amplified input genomic DNA fraction according to the formula: IP(Ct)/IN(Ct). The calculated immunoprecipitation enrichment was plotted and standard error bars were displayed.

### Sodium bisulfite mapping of DNA methylation

Sodium bisulfite mapping was performed as previously described[55]. After gene-specific PCR amplification (**Table S2**) of sodium bisulfite treated DNA for each subject, a mix of 10 ng of the gel-extracted PCR product from all of the subjects from each High and Low LG group (n = 3/group) were used for subsequent molecular cloning (Cequation 8800, Beckman-Coulter). We obtained 20 clones for sequencing from 2–3 independent PCR reactions for each subject.

### Genotyping

The genes verified for differences in DNA methylation by qCHIP analysis were further analyzed for genotyping using identical primers (**Table S2**). The resulting PCR products for each subject were sequenced bidirectionally using the forward and the reverse primer by Genome Quebec (ABI 3100, Applied Biosystems). Genetic variation was assessed throughout the PCR amplicon used for qCHIP analysis by alignment of genomic DNA with the published gene sequence (CLC Workbench, CLC bio).

### Quantitative real-time RT-PCR analysis

The expression patterns of 45 transcripts examined by microarray were quantified. For genes shown in **Figure 1c** (also see **Table S1**) primer design (**Table S2**) and analysis were performed by Genome Quebec (ABI lightcycler, ABI biosystems), whereby the expression of an additional 7 housekeeping genes (Actb, Gapdh, Gusb, Pum1, Rpl19, Rps18, Tubb5) was assessed for the same subjects used for microarray hybridization. The gene showing the least variance between High and Low LG adult offspring was selected as the reference gene for all subjects (GusB), and statistical significance, fold differences and standard errors of the mean were calculated according to published methods using the freely-available Relative Expression Software Tool program[56]. For the quantification of gene expression differences related to the *NR3C1* gene shown in **Figure 4b** and the Pcdh gene clusters shown in **Figure 5** (also see **Table S1**), a standard curve was generated from 7 serial dilutions of a mixture of cDNA from each High and Low LG offspring, and gene expression was quantified relative to the tubulin housekeeping gene (480 lightcycler, Roche) for an additional cohort of 8 High LG and 8 Low LG offspring, according to previously published methods (**Table S2**; [19], [57]). All reactions for all genes were performed in triplicate and statistical significance was determined as P<0.05 using one-tailed t-tests.

## Supporting Information

Methods S1 (0.08 MB PDF)

Table S1 Genes with higher expression in High LG offspring. Listed are the fold change for 44 transcripts selected for gene expression profiling (High LG/Low LG). Expression is significantly higher in the High LG offspring for 29 transcripts (** = P<0.01, * = P<0.05). Also shown are distances to the nearest RDme and RDac both before and after the transcription start site of each gene, and whether they are hyper-methylated/acetylated or hypo-methylated/acetylated in High relative to Low LG adult offspring.
(0.04 MB XLS)

Table S2 Sequence information for primers used for H3K9 acetylation, DNA methylation, and gene expression validation of microarrays.
(0.04 MB XLS)

Figure S1 Pearson correlation between DNA methylation levels estimated from microarray data and levels estimated from qChIP for each gene validated by quantitative real-time PCR (red circles).
(0.07 MB TIF)

Figure S2 DNA methylation validated by sodium bisulfite mapping showing expected enrichment of DNA methylation in Low (black bars) compared to High LG (white bars) animals for the majority of CpG sites examined. These data confirm the enrichment in Low LG relative to High LG offspring estimated from microarray and qChIP.
(0.10 MB TIF)

Figure S3 DNA methylation, H3K9 acetylation and gene expression levels. (a) Average levels of H3K9 acetylation and DNA methylation across all regions, and gene expression levels within protein coding exons only for all subjects are depicted across the 7Mb region centered at the NR3C1 gene (see Supporting Methods for calculation of levels). (b) Levels across gene-associated regions for all genes are depicted. (c) Levels are depicted across CpG islands (H3K9 acetylation levels are red, DNA methylation levels are blue, and gene expression levels are green). All data are mean values and line thickness denotes SEM.
(0.30 MB TIF)

Figure S4 An example of predicted nucleosome occupancy and actual H3K9 acetylation levels estimated from microarray data for Protocadherin-α genes. Predictions were obtained in silico solely from DNA sequence using a previously published tool [1].
(0.32 MB TIF)
