# Supplementary material for: Broad Epigenetic Signature of Maternal Care in the Brain of Adult Rats
Source: PLoS One. 2011 Feb 28;6(2):e14739. doi: 10.1371/journal.pone.0014739 (PMC3046141; doi:10.1371/journal.pone.0014739)
Supplement: Methods S1 — (0.08 MB PDF) [file pone.0014739.s001.pdf]

Supporting Methods

Maternal Behavior. The behavior of each dam was observed for six 72-min observation periods daily for the first 6 days postpartum. Observations occurred at regular times each day, with four periods during the light phase (1000, 1300, 1700, 1900 hours) and two periods during the dark phase of the L:D cycle (2000 and 0600 hours). Within each observation period, the behavior of each mother was scored every 3 min (25 observations per period X 6 periods per day = 150 observations per mother per day). All observations were performed by individuals unaware of the origin of the animals. On occasion, because of disturbances in the animal room, observation sessions were uncompleted. Although all mothers were observed for exactly the same number periods, there was some variation across days. The data were analyzed as the percentage of observations in which animals engaged in the target behavior. The following behaviors were scored: mother off pups, mother licking/grooming any pup, or mother nursing pups in an arched-back posture, a "blanket" posture in which the mother lies over the pups, or a passive posture in which the mother is lying on her back or side while the pups nurse. A detailed description of these behaviors is provided elsewhere [2]. Note that behavioral categories are not mutually exclusive. For, example, licking/grooming most often occurred while the mother was nursing the pups. The frequency of maternal licking/grooming and arched-back nursing across a large number of mothers is normally and not bimodally distributed [3]. Hence, the high and low LG mothers represent two ends of a continuum, rather than two distinct

populations. To define these populations for the current study we observed the maternal behavior in a cohort of 32 mothers and devised the group mean and standard deviation for each behavior over the first 7 days of life. High LG mothers were defined as females whose frequency scores for both licking/grooming and arched-back nursing were >1 SD above the mean. Low LG mothers were defined as females whose frequency scores for both licking/grooming and arched-back nursing were >1 SD below the mean. Previous reports [4, 5] of licking/grooming behavior suggest that the frequency of licking/grooming and arched-back nursing are highly correlated ($r > +0.90$).

Microarray analysis. Differential methylation, acetylation and expression between maternal care groups was determined in two stages to ensure both statistical significance and biological relevance. In the first stage, linear models implemented in the 'limma' package [6] of Bioconductor[7] were used to compute a modified t statistic at the individual probe level. An individual probe was called differentially methylated/acetylated/expressed if the significance of its t statistic was at most 0.05 (uncorrected for multiple testing) and the associated difference of means between the groups was at least 0.5. Given that the DNA samples were sonicated into 200-700bp fragments prior to hybridization, we assumed that probes within 500bp should have approximately similar probe scores. Therefore, in the second stage, we computed differential statistics for 1000bp intervals from the differential statistics of the probes that they contained. The intervals tiled the entire 7Mb region under investigation at 500bp spacing. Differential significance

of these intervals was determined using the Wilcoxon rank-sum test comparing t statistics of the probes within the interval against those of all the probes on the microarray. Significance levels were then adjusted to obtain false discovery rates. An interval was called differentially methylated/acetylated/expressed if it satisfied each of the following:

1. its false discovery rate was at most 0.2, and
2. the 1000 bp interval contained at least one probe called differentially methylated/acetylated/expressed.

The first requirement ensured that several probes in the interval have similar group differences, and the second requirement ensured that the difference was not simply weakly distributed across the entire interval and consequently difficult to validate. For methylation and acetylation, intervals satisfying these tests were called an RDme or RDac (differentially methylated/acetylated region, respectively). Consecutive RDme/RDac were combined into a single RDme/RDac.

Figure 2 shows the difference of probe scores between the High LG and Low LG samples across the approximately 7Mb region. The number of significant differences within each 100KB region were summed and plotted. The image was generated by saving the differences to UCSC wiggle track files and then uploading them for display on the UCSC genome browser (http://genome.ucsc.edu/).

Supporting Figure 3a shows overall levels across the same 7Mb estimated from the DNA methylation, H3K9 acetylation and RNA expression probe scores.

To estimate DNA methylation levels across the region from microarray data, we computed the average methylation probe score for each probe across all samples and replicates and then applied a Bayesian deconvolution approach called the Batman algorithm [8] to the resulting probe summaries. Deconvolution is necessary because each probe measurement is influenced by methylation levels at multiple nearby CG dinucleotides. To reduce the computational burden, we actually reduce estimate resolution from individuals CG dinucleotides to 50 bp intervals tiling the entire region with each interval representing all CG dinucleotides that it contains. For H3K9 acetylation, we modified the deconvolution algorithm to estimate H3K9 acetylation levels at regularly spaced intervals (50 bp) rather than cytosine methylation levels. For RNA expression, we simply shifted the probe scores to maximize the percentage of probes inside annotated exons with scores above 0 while minimizing the percentage of probes outside annotated exons with scores above 0. The image of Supporting Figure 3a was generated by saving the resulting levels to UCSC wiggle track files and then uploading them for display on the UCSC genome browser (http://genome.ucsc.edu/).

Supporting Figure 3b summarizes estimated methylation, H3K9 acetylation and RNA expression levels in various regions associated with genes as well as CpG islands. The region upstream of the gene summarizes estimated levels from -2000bp to the 5' end of each gene in the 7Mb region under investigation. The graphs show average levels calculated at 20 intervals evenly spaced across the 2000 bases. Line thickness illustrates the standard error about

the mean. The section of the graph showing the region downstream of the gene was calculated similarly. Exons and introns were handled similarly except that, in order to aid in aligning the ends of the exons and introns, their lengths were normalized so that each exon and intron had a length of 1.

Figure 3a illustrates the correlation of methylation and acetylation differences across various distances. More specifically, the graph shows the Pearson correlation of modified t-statistics computed by limma for all pairs of probes at given distances within a 10% tolerance. Error bars indicate the 95% confidence intervals for the correlations obtained from 1000 bootstraps composed of randomly selected probe pairs with replacement. The gray region contains the 95% confidence interval for correlations of probe pairs independent of distance. The interval was computed from 500 random permutations of the probe coordinates in order to simulate independence.

Figure 3b illustrates significance of RDme enrichment in various regions associated with genes (e.g. exons). Enrichment for a set of regions was measured as the number of bases contained inside both an RDme and one of the regions. Significance of the enrichment was computed against a background of enrichment measurements obtained from 10000 randomized RDme locations. The graphs are similar to those in Supporting Figure 3b except that enrichment significance is computed and plotted at 10 evenly spaced intervals across each type of region (e.g. exon, intron).

For data shown in Figure 3c, modified t-statistics (described above) are used for each probe rather than for estimated levels in order to illustrate the

distribution of DNA methylation, H3K9 acetylation and transcriptional differences across the gene.

Figures 4a, and 5a-b were created by viewing UCSC browser tracks containing average microarray probe intensity differences between LG offspring groups.

References

1. Kaplan, N., et al., *The DNA-encoded nucleosome organization of a eukaryotic genome.* Nature, 2009. **458**(7236): p. 362-6.
2. Myers, M.M., et al., *Maternal behavior of SHR rats and its relationship to offspring blood pressures.* Dev Psychobiol, 1989. **22**(1): p. 29-53.
3. Champagne, F.A., et al., *Variations in maternal care in the rat as a mediating influence for the effects of environment on development.* Physiol Behav, 2003. **79**(3): p. 359-71.
4. Levine, S., *The ontogeny of the hypothalamic-pituitary-adrenal axis. The influence of maternal factors.* Ann N Y Acad Sci, 1994. **746**: p. 275-88; discussion 289-93.
5. Meaney, M.J., *Maternal care, gene expression, and the transmission of individual differences in stress reactivity across generations.* Annu Rev Neurosci, 2001. **24**: p. 1161-92.
6. Smyth, G.K., *Limma: linear models for microarray data.*, in *Bioinformatics and Computational Biology Solutions using R and Bioconductor*, V.C. R.

Gentleman, S. Dudoit, R. Irizarry, W. Huber Editor. 2005, Springer,: New York. p. 397-420.

7. Gentleman, R.C., et al., *Bioconductor: open software development for computational biology and bioinformatics.* Genome Biol, 2004. **5**(10): p. R80.

8. Down, T.A., et al., *A Bayesian deconvolution strategy for immunoprecipitation-based DNA methylome analysis.* Nat Biotechnol, 2008. **26**(7): p. 779-785.
