# Supplementary figures and images for: Broad Epigenetic Signature of Maternal Care in the Brain of Adult Rats
Source: PLoS One. 2011 Feb 28;6(2):e14739. doi: 10.1371/journal.pone.0014739 (PMC3046141; doi:10.1371/journal.pone.0014739)

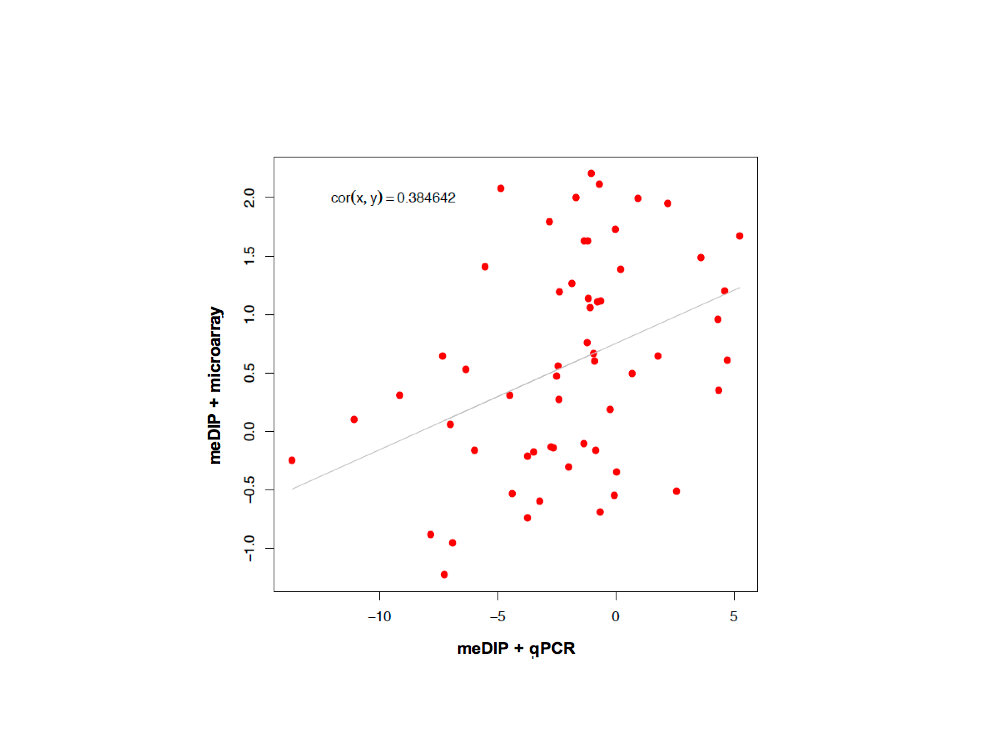

Supplement: Figure S1 — Pearson correlation between DNA methylation levels estimated from microarray data and levels estimated from qChIP for each gene validated by quantitative real-time PCR (red circles). (0.07 MB TIF) [file pone.0014739.s004.tif]

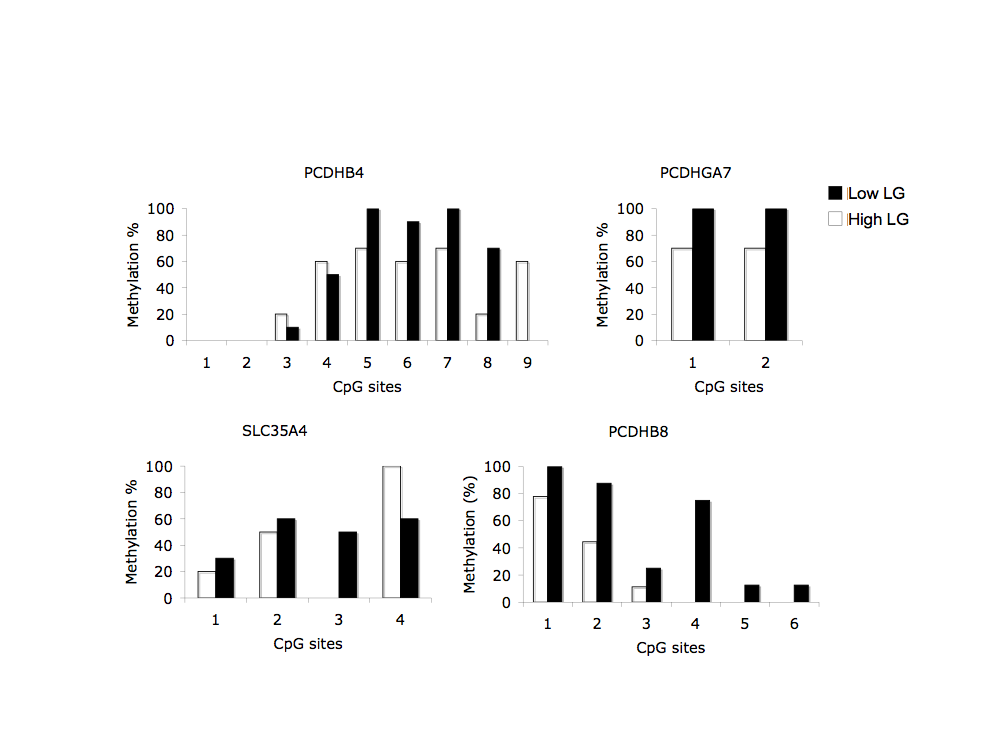

Supplement: Figure S2 — DNA methylation validated by sodium bisulfite mapping showing expected enrichment of DNA methylation in Low (black bars) compared to High LG (white bars) animals for the majority of CpG sites examined. These data confirm the enrichment in Low LG relative to High LG offspring estimated from microarray and qChIP. (0.10 MB TIF) [file pone.0014739.s005.tif]

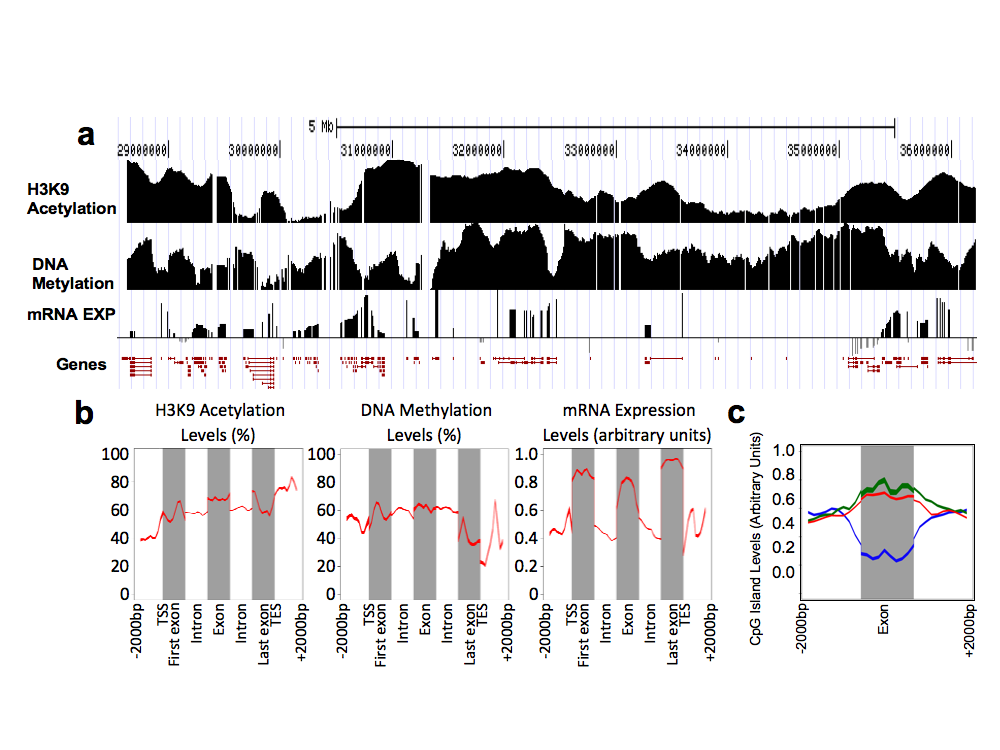

Supplement: Figure S3 — DNA methylation, H3K9 acetylation and gene expression levels. (a) Average levels of H3K9 acetylation and DNA methylation across all regions, and gene expression levels within protein coding exons only for all subjects are depicted across the 7Mb region centered at the NR3C1 gene (see Supporting Methods for calculation of levels). (b) Levels across gene-associated regions for all genes are depicted. (c) Levels are depicted across CpG islands (H3K9 acetylation levels are red, DNA methylation levels are blue, and gene expression levels are green). All data are mean values and line thickness denotes SEM. (0.30 MB TIF) [file pone.0014739.s006.tif]

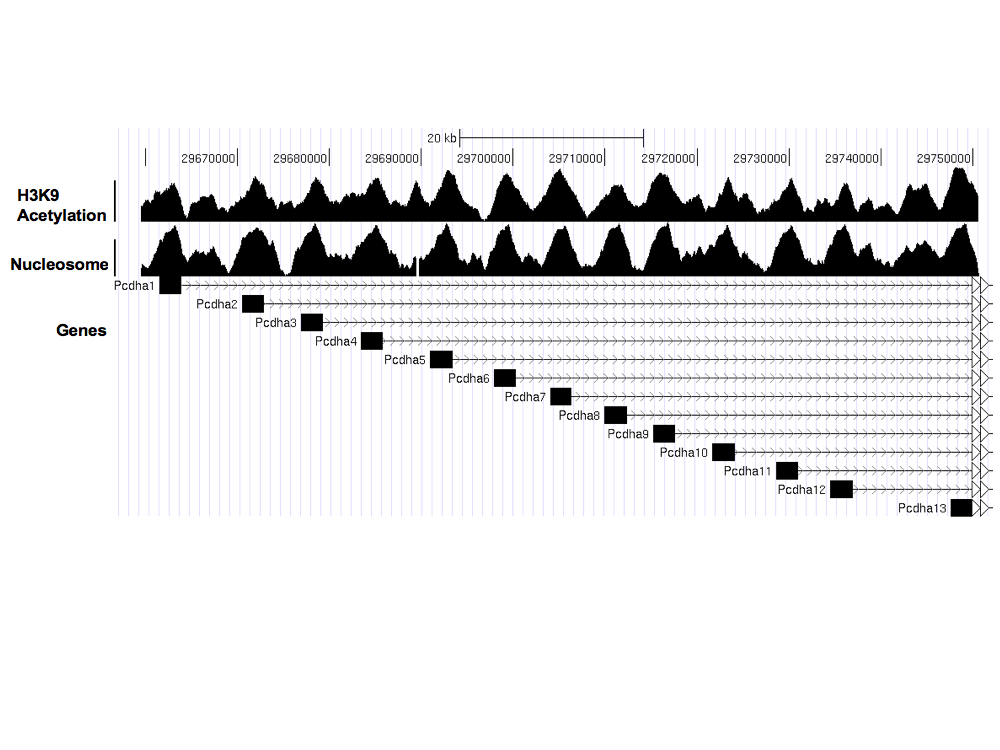

Supplement: Figure S4 — An example of predicted nucleosome occupancy and actual H3K9 acetylation levels estimated from microarray data for Protocadherin-α genes. Predictions were obtained in silico solely from DNA sequence using a previously published tool [1]. (0.32 MB TIF) [file pone.0014739.s007.tif]
